# Effects of Multimodal Physical Therapy on Pain, Disability, H-reflex, and Diffusion Tensor Imaging Parameters in Patients With Lumbosacral Radiculopathy Due to Lumbar Disc Herniation: A Preliminary Trial

**DOI:** 10.7759/cureus.63501

**Published:** 2024-06-30

**Authors:** Mohamed Badr, Hosny Elkhawaga, Khaled Fawaz, Mohamed Kasem, Eman Fayez

**Affiliations:** 1 Department of Physical Therapy for Neuromuscular Disorders and its Surgery, Faculty of Physical Therapy, Cairo University, Giza, EGY; 2 Department of Physical Therapy for Neuromuscular Disorders and its Surgery, Faculty of Physical Therapy, Al Hayah University in Cairo, Cairo, EGY; 3 Faculty of Physical Therapy, Cairo University, Giza, EGY; 4 Department of Orthopedic Surgery, Faculty of Medicine, Cairo University, Giza, EGY; 5 Department of Diagnostic and Interventional Radiology, Faculty of Medicine, Cairo University, Giza, EGY

**Keywords:** diffusion tensor imaging (dti), manual therapy methods, low-back pain (lbp), apparent diffusion coefficient (adc), fractional anisotropy, h-reflex, physiotherapy intervention, physical therapy rehabilitation, lumbar disc herniation (ldh), lumbosacral radiculopathy

## Abstract

Background

Lumbosacral radiculopathy (LSR) due to lumbar disc herniation (LDH) is a condition caused by mechanical compression of nerve roots. Various physical therapy interventions have been proposed for the conservative management of LSR due to LDH. However, the study of physical therapy interventions in a multimodal form is lacking. Additionally, the effect of physical therapy on diffusion tensor imaging (DTI) parameters of the compressed nerve root has not been studied. This study aimed to investigate the effects of multimodal physical therapy (MPT) on pain, disability, soleus H-reflex, and DTI parameters of the compressed nerve root in patients with chronic unilateral LSR due to LDH.

Methods

A prospective preliminary pre-post clinical trial with a convenience sample was conducted. A total of 14 patients with chronic unilateral LSR due to paracentral L4-L5 or L5-S1 LDH were recruited for the study. Participants received a total of 18 sessions of a six-week MPT program that consisted of electrophysical agents, manual therapy interventions, and core stability exercises. Electrophysical agents involved interferential current and hot pack. Manual therapy interventions included myofascial release, side posture positional distraction, passive spinal rotation mobilization, and high-velocity low-amplitude manipulation. Visual analog scale (VAS), Roland-Morris Disability Questionnaire (RMDQ), soleus H-reflex amplitude, side-to-side amplitude (H/H) ratio, fractional anisotropy (FA), and apparent diffusion coefficient (ADC) of the compressed nerve root were measured at baseline and post-intervention.

Results

There were significant improvements in VAS, RMDQ, H/H ratio, FA, and ADC of the compressed nerve root. Furthermore, significant improvement was found in the affected side compared with the contralateral side in H-reflex amplitude.

Conclusions

The observations of this preliminary trial suggest that MPT is a successful intervention in patients with chronic unilateral LSR due to LDH. Regarding DTI parameters of the compressed nerve root, FA increased and ADC decreased. Future studies with a control group, large sample sizes, and longer follow-up periods are needed.

## Introduction

Lumbosacral radiculopathy (LSR) is a condition caused by mechanical compression of nerve roots and characterized by sensory, motor, and/or reflex changes [1]. LSR affects approximately 3% to 5% of the general population [2], with an incidence of 4.86 per 1000 person-years [3]. Lumbar disc herniation (LDH) is the major cause of LSR. However, other causes include spinal stenosis, inflammatory disorders, tumors, and infection [2].

LSR due to LDH is diagnosed by a combination of history, physical examination, and magnetic resonance imaging (MRI) findings [4]. The soleus H-reflex is a useful neurophysiological procedure for the diagnosis of LSR [4,5]. The diagnostic criteria have been reported to be side-to-side amplitude (H/H) ratios smaller than 0.5 in the presence of side-to-side latency differences of more than 1.0 ms, H-reflex latency of more than 30 ms, or absence of the H-reflex on the affected side [6]. Additionally, Alrowayeh and Sabbahi, 2011 found that the H/H ratio was 0.67 when the latency was normal, and it reduced to 0.54 when the latency was abnormal in patients with LSR [7].

Diffusion tensor imaging (DTI) is an MRI technique that can analyze the diffusion of water molecules in living tissues and reflect their internal microstructural characteristics [8]. This technique has been used to evaluate pathological changes in the brain, spinal cord, and peripheral nervous system [9]. Fractional anisotropy (FA) and apparent diffusion coefficient (ADC) are important parameters of DTI [8]. Patients with LSR due to LDH demonstrated a decrease in FA and an increase in ADC values of the compressed nerve root [9].

The management of LSR due to LDH varies from medications to physical therapy, injections, and surgery [10]. Several physical therapy interventions have been proposed for the conservative management of LSR due to LDH. Physical therapy interventions include manual therapy, therapeutic exercise, electrophysical agents, and patient education [11]. However, the study of physical therapy interventions in a multimodal form is lacking. Moreover, pain and disability are the most frequently reported outcome measures of treatment efficacy, and objective measures such as H-reflex and DTI parameters have not been intensively assessed in studies of the physical therapy management of LSR due to LDH [12-14]. To the best of our knowledge, the effect of physical therapy on DTI parameters of the compressed nerve root has not been studied. Therefore, the aim of this study was to investigate the effects of a multimodal physical therapy (MPT) program consisting of electrophysical agents, manual therapy interventions, and core stability exercises on pain, disability, soleus H-reflex, and DTI parameters of the compressed nerve root in patients with chronic unilateral LSR due to LDH.

## Materials and methods

Study design, ethics, and setting

A prospective preliminary pre-post clinical trial with a convenience sample was conducted. The study was performed in line with the principles of the Declaration of Helsinki and written informed consent was obtained from all participants. The Research Ethics Committee of the Faculty of Physical Therapy, Cairo University approved all procedures (No: P.T.REC\012\001986). This trial was retrospectively registered in the ClinicalTrials.gov with NCT06058806 registration number (28/09/2023). The study was conducted in the outpatient clinic, Faculty of Physical Therapy, Cairo University in the period from June 2018 to Jan 2020.

Participants

Patients were diagnosed with LSR and referred by a physician before screening for eligibility. Participants were included if they met the following criteria: (1) chronic (≥ 3 months) unilateral LSR due to paracentral L4-L5 or L5-S1 intervertebral disc herniation confirmed radiologically by MRI, clinically by history and physical examination, and neurophysiological by soleus H-reflex assessment [4], (2) presence of at least one of the following findings regarding H-reflex: H/H ratios smaller than 0.5 in the presence of side-to-side latency differences of more than 1.0 ms, H-reflex latency of more than 30 ms [6], or H/H ratio smaller than 0.67 in the absence of latency difference [7], (3) body mass index less than 30, and (4) 20-45 years of age.

Participants were excluded if they had any of the following criteria: (1) systemic diseases such as autoimmune and metabolic diseases, (2) previous surgeries, (3) steroid injection, (4) lumbar spinal stenosis, (5) spinal deformity, (6) spinal fracture, (7) bilateral symptoms, (8) radiological evidence of bilateral nerve root encroachment even if asymptomatic, or (9) evidence of H-reflex amplitude or latency affection on the asymptomatic side.

Interventions

Each patient received 18 sessions over a six-week period (three sessions per week). MPT program consisting of electrophysical agents, manual therapy interventions, and core stability exercises was delivered to the patients by a physical therapist with more than seven years of experience in spine rehabilitation under the supervision of a physical therapy consultant with more than 20 years of experience in the management of spinal conditions.

Electrophysical agents: The patient was positioned prone with a pillow under the pelvis. Interferential current (IFC) was applied at the lumbosacral region along with a hot pack over the low back region for 20 min. The interferential therapy was delivered with a frequency of 4 KHz modulated at 100 Hz [15].

Manual therapy interventions: Following electrophysical agents, myofascial release was performed on low back muscles and gluteus, piriformis, hamstring, and calf of the affected side [16]. Then, the patient was given side posture positional distraction [17,18]. The patient was positioned side-lying on a roll placed under the affected level of the lumbar spine, with the affected side directed upwards and the pain-free side directed downwards. The affected leg was maintained in flexion at the hip and knee, and the pain-free leg was maintained in slight flexion. The patient was maintained in this position for 20 minutes. Following positional distraction, the roll was removed and the patient was maintained in the same position. The physical therapist performed passive spinal rotation mobilization on the affected side [19,20]. The physical therapist stood behind the patient, stabilizing the uppermost shoulder while pushing the pelvis. Finally, high-velocity low-amplitude (HVLA) manipulation was applied from the same position [21,22].

Core stability exercises: Following manual therapy, patients received a program of core stability exercises, which included side plank on knee or ankle on both sides [23], prone plank on knee or ankle, abdominal curl up [23], 60-degree hip flexion clamshell with thera-band with the affected leg directed upwards [24], and hip abductors and extensors of the affected side strengthening from standing with thera-band [25]. Each exercise was performed in three sets of 10 repetitions except side plank and plank, which was performed three times in a static manner for 10-20 seconds or up to patient failure. The rest interval was 30 seconds between the sets and one minute between the exercises. The exercise program was individualized according to patient irritability and tolerability in the sessions. The patients began with clamshell, side plank on the knee, and prone plank on the knee in the first sessions. After this, they progressed to plank and side plank on the ankle, abdominal curl up, and hip abductors and extensors strengthening from standing.

Outcome measures

All of the outcome measurements were assessed at two time points: at baseline and six weeks post-intervention.

Pain: A visual analog scale (VAS) was used to assess the intensity of back pain. It is a horizontal 10 cm line ranging from 0 cm (no pain) to 10 cm (worst pain). The minimal clinically important difference (MCID) of the VAS has been reported to be 2 cm in patients with chronic low back pain (CLBP) [26].

Disability: Roland-Morris Disability Questionnaire (RMDQ) was used to measure the level of disability. It is a self-reported questionnaire with a score ranging from 0 (no disability) to 24 (worst disability). We used the Arabic version of the RMDQ, which is valid and reliable [27]. The MCID of the RMDQ has been reported to be 3.5 points [26].

Soleus H-reflex assessment: Neurosoft EMG unit (Neuro-MEP-Micro version 2009, Russia) was used for soleus H-reflex assessment. H-reflex amplitude, H-reflex latency, H/H ratio, and side-to-side latency difference of each patient were calculated for inclusion criteria, and H-reflex amplitude and H/H ratio were taken as outcome measurements. The H/H ratio was calculated by dividing the H-reflex amplitude value of the affected side by the non-affected side [6]. Regarding H-reflex stimulation and recording, we used the same method described in the previous studies [5,7]. The patient was lying in the prone position with the head in the neutral position. The active surface bar recording electrode was placed 3 cm distally to the bifurcation of the gastrocnemii, and the reference recording electrode was placed 2 cm distally. A silver-silver chloride surface stimulating bar electrode was used to stimulate the tibial nerve in the popliteal fossa, with a percutaneous electrical stimulus of 1.0 ms duration at a frequency of 0.2 pps. The cathode of the stimulating electrode was proximal to the anode to avoid the anodal block. A 2 cm-diameter ground metal electrode was applied between the stimulating and the recording electrodes. Five traces of the maximum H-reflex were elected, averaged, and included in the analysis.

DTI parameters: FA and ADC of the compressed nerve root were assessed. All patients underwent a 1.5 Tesla MRI examination (MAGNETOM Aera, Siemens Healthcare, Germany) in the supine position. The field gradient and slew rate were 33 mT/m and 125 T/m/s, respectively. Sixty elements body phased array coil was used. Coronal fat suppressed single-shot fast-spin-echo (FSE) echo-planar (EPI) DTI sequence with 20 encoded directions was used. In addition, diffusion-weighted images with b values of 500 and 1000 s/mm^2^ and b0 images per DTI sequence were applied. The image acquisition parameters were: repetition time (TR) = 7700 ms, echo time (TE) = 97 ms, field of view (FOV) = 350 x 100, slice count = 49, slice thickness = 2 mm with gap zero, calculated voxel size = 2.7 x 2.7 x 2 mm^3^. The total acquisition sequence time was eight minutes and 10 seconds. After DTI sequence acquisition, acquired data were transferred to a workstation with specialized software (Syngo.via, Neuro3D, Siemens Healthcare, Germany) for quantitative assessment of DTI sequence and fibers tracking. The DTI tractography was performed by automatic tracing, with tractography parameters as follows: seed points per voxel length of two, angle threshold of 30, and FA threshold of 0.2. The region of interest (ROI) was placed on the compression site of the affected nerve root. ROI location on the color-coded coronal planes was confirmed using T2 weighted coronal, axial, and sagittal images included in the standard MRI protocol. FA and ADC values for each ROI were automatically calculated.

Statistical analysis

The IBM SPSS version 25 (IBM Corp., Armonk, USA) was used for statistical analysis. The baseline participant characteristics were summarized with means ± standard deviation and range for quantitative variables (age, height, weight, and body mass index (BMI)) and frequency for qualitative variables (gender and affected level). The Shapiro-Wilk test was used for each variable to test the normality of distribution and revealed that all variables were normally distributed. Therefore, a paired t-test was used to compare post-intervention with the baseline and affected side with the contralateral side. Effect sizes for all variables were calculated using Cohen’s d. Cohen’s d indications are 0.2 = small effect, 0.5 = moderate effect, and 0.8 ≥ large effect with very significant clinical relevance. The alpha level was set at 0.05.

## Results

Participants

**Figure 1:**
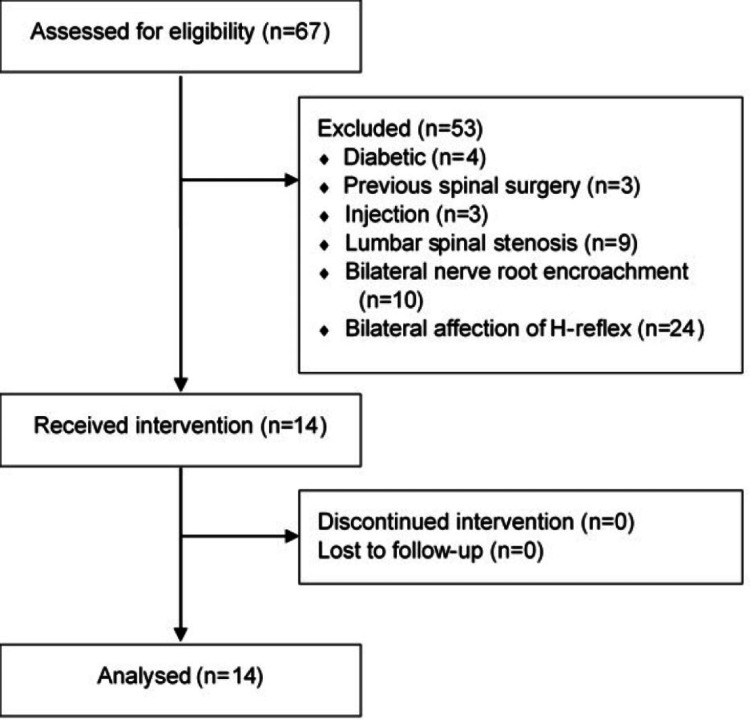
Flow chart of study participants

A total of 67 patients were assessed for eligibility. Of these, 53 did not meet the inclusion criteria. This resulted in 14 patients (nine males and five females) with LSR due to LDH who received the intervention and were analyzed. Figure 1 shows the study recruitment and procedure.

The mean age of the patients was 27±6.01 years. The mean body mass index was 25.36±1.8 kg/m^2^. Baseline characteristics of the patients are presented in Table 1.

**Table 1:** Baseline participant characteristics (n=14) BMI: body mass index

	Mean±SD	Range
Age (years)	27±6.01	(20–39)
Height (cm)	170.5±10.41	(152–187)
Weight (kg)	74.07±11.26	(60–93)
BMI (kg/m^2^)	25.36±1.8	(22.84–28.08)
Gender, n (%)		
Male	9 (64.29)	
Female	5 (35.7)	
Affected level, n (%)		
L4-L5	5 (35.7)	
L5-S1	9 (64.29)	

Outcomes

Overall, there were improvements in pain (VAS), disability (RMDQ), H/H ratio, FA, and ADC of the compressed nerve root. Furthermore, there were improvements in the affected side compared with the contralateral side in H-reflex amplitude. The means of the outcome measures at baseline and post-intervention and mean changes from baseline to post-intervention are presented in Table 2.

**Table 2:** Changes in outcome measures from baseline to post-intervention (n=14) Data are expressed as mean ± standard deviation and mean difference (95% confidence interval). ^*^ Indicates significant differences (p<0.05) ADC: Apparent diffusion coefficient; FA: Fractional anisotropy; H/H ratio: side-to-side H-reflex amplitude ratio; RMDQ: Roland-Morris Disability Questionnaire; VAS: Visual analog scale

	Baseline	Post-intervention	Change from baseline to post-intervention		Change in affected side versus contralateral side		
			MD (95% CI); p-value	Cohen’s d	MD (95% CI); p-value		Cohen’s d
VAS (0–10)	8.21±0.87	2.46±1.08	-5.75 (-6.47, -5.03); <0.001^*^	-4.61			
RMDQ (0–24)	17.57±3.27	4.64±2.5	-12.93 (-14.99, -10.87); <0.001^*^	-3.63			
H-reflex amplitude (mV)							
Affected side	1.5 ± 1.05	4.86±1.49	3.36 (2.6, 4.13); <0.001^*^	2.54	2.79 (1.84, 3.75); <0.001^*^		1.69
Contralateral side	5.24±1.47	5.81±1.85	0.57 (0.13, 1.01); 0.01^*^	0.75			
Affected side versus contralateral side, MD (95% CI; p-value)	-3.74 (-4.43, -3.04); <0.001^*^	-0.94 (-1.53, -0.36); 0.004^*^					
H/H ratio	0.27±0.16	0.85±0.13	0.58 (0.43, 0.73); <0.001^*^	2.29			
FA	0.226±0.018	0.268±0.012	0.043 (0.035, 0.05); <0.001^*^	3.27			
ADC (10^−3 ^mm^2^/s)	1.764±0.1	1.616±0.105	-0.147 (-0.199, -0.095); <0.001^*^	-1.64			

For pain, the mean VAS was 8.21±0.87 cm at baseline and 2.46±1.08 cm post-intervention, indicating a decrease of -5.75 cm (95% CI -6.47, -5.03; P<0.001) after the intervention. For disability, the mean RMDQ was 17.57±3.27 points at baseline and 4.64±2.5 points post-intervention, indicating a decrease of -12.93 points (95% CI -14.99, -10.87; P<0.001) after the intervention.

For H-reflex amplitude, a statistically significant decrease of -3.74 mV (95% CI -4.43, -3.04; <0.001) was found in the affected side compared to the contralateral side at baseline. Post-intervention, the mean change from baseline to post-intervention of H-reflex amplitude was 3.36 mV (95% CI 2.6, 4.13) in the affected side and 0.57 mV (95% CI 0.13, 1.01) in the contralateral side, indicating an increase of 2.79 mV (95% CI 1.84, 3.75; P<0.001) in the affected side. For H/H ratio, the mean was 0.27±0.16 at baseline and 0.85±0.13 post-intervention, indicating an increase of 0.58 (95% CI 0.43, 0.73; P<0.001) after the intervention.

For DTI parameters of the compressed nerve root, the mean FA was 0.226±0.018 at baseline and 0.268±0.012 post-intervention, indicating an increase of 0.043 (95% CI 0.035, 0.05; P<0.001) after the intervention. The mean ADC was 1.764±0.1 × 10​​​^−3^ mm^2^/s at baseline and 1.616±0.105 × 10^−3^ mm^2^/s post-intervention, indicating a decrease of -0.147 × 10^−3^ mm^2^/s (95% CI -0.199, -0.095; P<0.001) after the intervention.

## Discussion

This study assessed the effects of MPT on pain, disability, soleus H-reflex, and DTI parameters of the compressed nerve root in patients with chronic unilateral LSR due to LDH. We found that MPT was effective, with significant improvements in pain, disability, soleus H-reflex amplitude, H/H ratio, FA, and ADC.

Patients received an MPT program that consisted of electrophysical agents, manual therapy interventions, and core stability exercises. IFC has been reported to have an immediate effect on pain in patients with CLBP [15]. Franco et al. 2018 [28] demonstrated that the IFC prior to pilates exercises reduced pain faster than the placebo IFC in patients with CLBP. Electrophysical agents were applied for pain modulation and preparation of patients before manual therapy and therapeutic exercises.

Regarding manual therapy interventions, A meta-analysis by Wu et al. 2021 [16] showed that myofascial release improves pain and function in patients with CLBP. While Creighton, 1993 [18] confirmed radiologically that positional distraction opens the intervertebral foramen, Mitchell et al. 2001 [17] showed that positional distraction has immediate improvements in the centralization of leg pain and straight leg raising height in patients with unilateral LSR. Additionally, Cramer et al. 2013 [29] demonstrated that side-posture positioning increases lumbar zygapophyseal joint space separation and adds therapeutic benefit to manipulation in patients with low back pain. Passive spinal rotation mobilization and HVLA manipulation were applied to increase mobility, stimulate mechanoreceptors and proprioceptors in nearby structures, and open the intervertebral foramen to reduce pressure over the sensitized nerve root [30]. Previous studies showed that HVLA manipulation was effective for patients with LSR [21,22].

Finally, core stability exercises were performed to increase stability in the lumbopelvic region and improve muscle activation, which has been reported to be changed in low back pain [31]. Moreover, Pourahmadi et al. 2014 [14] concluded that core stability exercise effectively decreases pain and disability in patients with symptomatic LDH.

Several physical therapy studies assess the individual effect of a specific intervention and lack MPT [12-14]. However, in clinical practice, physical therapy should address pain, mobility deficits, movement coordination impairments, and neural components. Therefore, individualized MPT should be recommended according to the patient’s condition. In our study, we used MPT to investigate the overall effect of all used interventions. MPT resulted in a pain reduction of 5.5 cm as measured by the VAS, which is more than the MCID of 2 cm [26]. Furthermore, disability, as measured by the RMDQ, decreased by 12.93 points, which is more than the MCID of 3.5 points [26]. Regarding H/H ratio, it increased from 0.27 to 0.85, which is similar to the H/H ratio value of 0.83 reported in healthy people by Alrowayeh and Sabbahi, 2011 [7].

Regarding DTI parameters of the compressed nerve root, Wang et al. 2022 [9] demonstrated that FA decreases and ADC increases in patients with nerve damage due to LDH. Our findings showed that MPT increased FA and decreased ADC. Although there is no previous study to investigate the effect of MPT on DTI parameters of the compressed nerve root in LSR, several studies investigated the effect of surgical intervention [32-35]. They found that FA decreases after surgical intervention [32-35], which is in agreement with our results. Regarding ADC, it remains controversial whether it decreases [32,33] or does not change [34,35].

Strengths

Patient selection in our study was very limited to confirmed LSR due to LDH patients with clinical, radiological, and neurophysiological assessments. The intervention in the study was in a multimodal form to combine the overall effect of all used interventions and reflect the physical therapy effect in clinical practice. In addition to pain and disability, our study addressed the neurophysiological H-reflex and DTI parameters. Of note, this is the first study to assess the effect of physical therapy on DTI parameters of the compressed nerve root in LSR.

Limitations and recommendations

The small sample size and lack of a control group and follow-up are limitations. Although our study had no control group, we compared the affected side with the contralateral healthy side of the same patient regarding H-reflex amplitude. Further, high-quality randomized controlled trials with larger sample sizes and longer duration of follow-up are recommended.

## Conclusions

The observations of this preliminary trial suggest that MPT is a successful intervention in patients with chronic unilateral LSR due to LDH. Pain, disability, and H-reflex amplitude were improved after six weeks of MPT. Regarding DTI parameters of the compressed nerve root, FA increased and ADC decreased.
